# Quantum Neural Network Based Distinguisher on SPECK-32/64

**DOI:** 10.3390/s23125683

**Published:** 2023-06-18

**Authors:** Hyunji Kim, Kyungbae Jang, Sejin Lim, Yeajun Kang, Wonwoong Kim, Hwajeong Seo

**Affiliations:** Division of IT Convergence Engineering, Hansung University, Seoul 02876, Republic of Korea

**Keywords:** quantum neural network, SPECK-32/64, differential, distinguisher

## Abstract

As IoT technology develops, many sensor devices are being used in our life. To protect such sensor data, lightweight block cipher techniques such as SPECK-32 are applied. However, attack techniques for these lightweight ciphers are also being studied. Block ciphers have differential characteristics, which are probabilistically predictable, so deep learning has been utilized to solve this problem. Since Gohr’s work at Crypto2019, many studies on deep-learning-based distinguishers have been conducted. Currently, as quantum computers are developed, quantum neural network technology is developing. Quantum neural networks can also learn and make predictions on data, just like classical neural networks. However, current quantum computers are constrained by many factors (e.g., the scale and execution time of available quantum computers), making it difficult for quantum neural networks to outperform classical neural networks. Quantum computers have higher performance and computational speed than classical computers, but this cannot be achieved in the current quantum computing environment. Nevertheless, it is very important to find areas where quantum neural networks work for technology development in the future. In this paper, we propose the first quantum neural network based distinguisher for the block cipher SPECK-32 in an NISQ. Our quantum neural distinguisher successfully operated for up to 5 rounds even under constrained conditions. As a result of our experiment, the classical neural distinguisher achieved an accuracy of 0.93, but our quantum neural distinguisher achieved an accuracy of 0.53 due to limitations in data, time, and parameters. Due to the constrained environment, it cannot exceed the performance of classical neural networks, but it can operate as a distinguisher because it has obtained an accuracy of 0.51 or higher. In addition, we performed an in-depth analysis of the quantum neural network’s various factors that affect the performance of the quantum neural distinguisher. As a result, it was confirmed that the embedding method, the number of the qubit, and quantum layers, etc., have an effect. It turns out that if a high-capacity network is needed, we have to properly tune properly to take into account the connectivity and complexity of the circuit, not just by adding quantum resources. In the future, if more quantum resources, data, and time become available, it is expected that an approach to achieve better performance can be designed by considering the various factors presented in this paper.

## 1. Introduction

As IoT technology develops, numerous sensor devices are being used in real life. Accordingly, a large amount of sensor data are being generated. However, if the personal data of sensor devices connected to the IoT network are leaked, it may cause great personal and social problems. Therefore, lightweight encryption technologies are applied to protect such sensor data, since the deployed sensors are often resource-constrained (low-cost, low-energy, etc.) [1]. SPECK-32 is a lightweight block cryptographic algorithm that is suitable for operation on sensor devices because it uses 32-bit plaintext and ciphertext and a 64-bit key [2]. However, attack techniques for lightweight cipher on sensor devices are also being studied [3].

A block cipher has a characteristic in which an output difference according to an input difference exists probabilistically. In addition, these differential characteristics are propagated and can be probabilistically predicted even when encryption is performed. That is, since a differential characteristic exists with a certain probability and can be predicted probabilistically, deep learning is suitable as a solution for this. Since Gohr’s work [4], numerous studies on deep-learning-based distinguishers have been conducted [5,6,7,8,9,10,11]. In addition, as quantum computers have been developed in recent years, a quantum neural network utilizing a quantum computer is attracting attention. Quantum neural networks replace the training process of classical neural networks with quantum circuits. In other words, the quantum circuit acts as a neural network. Simply, we use the rotation gate of a quantum circuit to update the output value of the circuit by changing the state of the qubit. This is similar to the training process in which classical neural networks reduce losses while updating the weights of the network. Quantum neural networks have the advantage of being able to achieve fewer parameters and higher performance compared to classical neural networks. However, since current quantum computers are noisy intermediate-scale quantum computers (https://horizon.kias.re.kr/16769/ accessed on 15 June 2023) (NISQs), it is difficult to correct errors in qubits. Therefore, a hybrid neural network that combines a classical neural network and a quantum neural network is now stable in terms of performance.

In this paper, we propose a novel distinguisher using quantum neural networks. Our quantum neural distinguisher distinguishes cryptographic data from random data. In a nutshell, when we input random or cryptographic data into our quantum neural distinguisher, it predicts which type of data they are. Furthermore, as described above, quantum neural networks can achieve higher performance and efficiency than classical neural networks in certain problems. Therefore, we proposed various approaches to design an effective quantum neural network based distinguisher. Our contributions are as follows.

### 1.1. Our Contribution

#### 1.1.1. Quantum Neural Network Based Distinguisher for 5-Round SPECK-32 in NISQ

In this work, we proposed a quantum neural network based distinguisher for SPECK-32 that can be feasible in NISQ. To the best of our knowledge, the first quantum neural network-based analysis of a modern block cipher. In NISQs, it is difficult to obtain quantum advantages over classical neural networks unless it is a simple task. However, for 5-round SPECK-32, our quantum neural distinguisher successfully distinguished cipher from random.

#### 1.1.2. In-Depth Analysis of Factors Affecting the Performance of the Quantum Neural Distinguisher

The performance of quantum neural networks is determined by the expressiveness and connectivity of quantum circuits, and they change due to various factors, such as the number of qubits and quantum layers in quantum circuits, the embedding method, and the number of parameters. In this paper, we performed experiments on various elements constituting quantum neural networks and conducted an in-depth analysis of them.

The remainder of this paper is organized as follows. In Section 2, the backgrounds of the neural distinguisher and the quantum neural network are covered. In Section 3, we present a quantum neural network based distinguisher for 5-round SPECK-32. In Section 4, the performance of the quantum neural distinguisher was evaluated and various factors affecting the performance of the quantum neural network were analyzed. Finally, Section 5 concludes the paper.

## 2. Background

### 2.1. Classical Neural Networks

**Figure 1:**
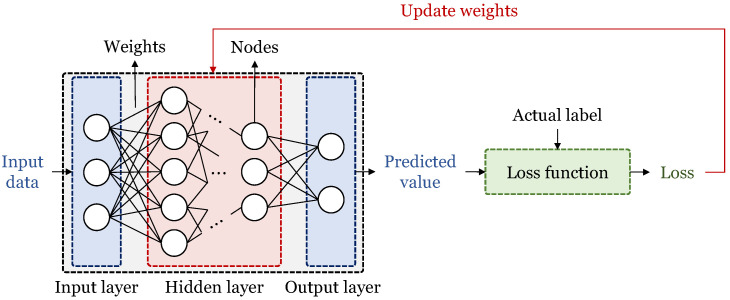
Training process of artificial neural network.

Artificial neural networks [12] are supervised learning algorithms. As shown in Figure 1, a neural network is constructed in the form of stacked layers of multiple nodes, and multiple layers that exist between the input layer and the output layer are called hidden layers. Neurons (nodes) of each layer are multiplied by weights and the values of neurons in the previous layer connected to them, and then they are added [13]. The calculated values are input to the nonlinear activation function, then the values are determined as the value of the current node. All nodes of the corresponding layer are input to the next layer and are used to calculate the values of the nodes. After performing this process in all layers, the final value of the network is output. The output is input to the loss function along with the label (the correct answer of the actual data). A loss is then calculated that represents the difference between the label and the predicted value. In order to minimize the loss, the weights of the neural network are updated, and the neural network is trained to make a correct prediction. Artificial neural networks can solve classification and regression problems. Classifying into two classes is called binary classification, and classifying into multiple classes is multiple classification. In addition, for this task, various structures of neural networks can be used. There are Multi-Layer Perceptron (MLP) with the most basic structure, Convolutional Neural Networks (CNNs) [14] effective for image processing, Recurrent Neural Networks (RNNs) [15] suitable for processing time series data, and Generative Adversarial Networks (GANs) [16], etc.

### 2.2. Quantum Neural Networks

#### 2.2.1. Quantum Circuit

Quantum circuits are constructed through bits and gates like classical logic circuits. However, instead of the classic bits and gates, quantum circuits use qubits and quantum gates that have the superposition and entanglement principles of quantum mechanics, and it works in quantum computers. A qubit is like a classical bit, but through superposition states, values exist as probabilities and are determined to be a single value after observation. In addition, since qubits can represent any value in the Bloch sphere, they can represent a richer range of values than classical bits. In addition, multiple physical qubits are required for one logical qubit (without error), and error correction techniques are required. However, current quantum computers do not have enough resources and techniques to correct errors.

**Figure 2:**
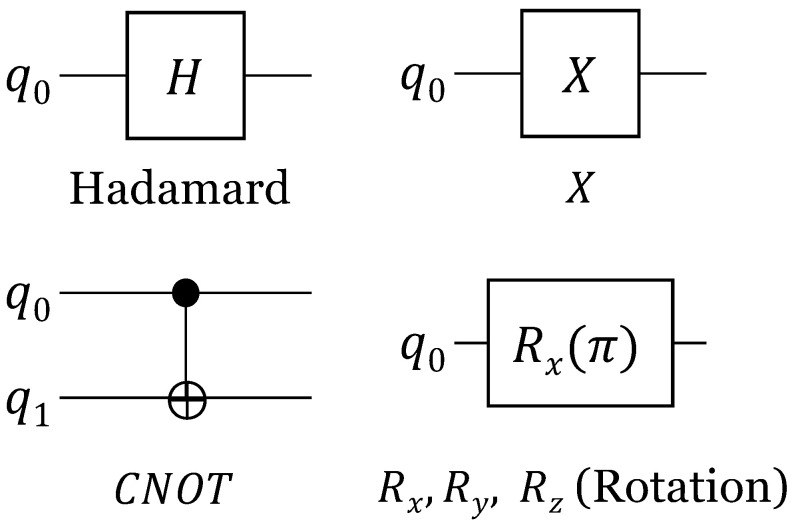
Quantum gates.

There are several types of gates used in quantum circuits [17], and the state of a qubit can be changed by applying quantum gates. Figure 2 shows some of the quantum gates. The Hadamard (*H*) gate makes a superposition so that qubits can have both 0 and 1 states at the same time. Furthermore, when the same qubit passes through the Hadamard gate again, it is restored to its original state. The *X* gate changes the state of the qubit (e.g., from 0 to 1), and it changes the probability in the superposition state. The CNOT gate uses the first qubit as the control qubit. When the control qubit is 1, the NOT operation is applied to the second qubit. Here, entanglement for two qubits is used. Next, there are Rx, Ry, and Rz rotation gates that rotate the qubits about the *x*, *y*, and *z* axes. These gates rotate the qubit by the rotation angle based on a specific axis.

Quantum circuits can be constructed by applying these quantum gates to qubits. At this time, an efficient circuit design is required considering the depth (length of the circuit) and width (the number of qubits) of the circuit. Quantum circuits can be implemented using the quantum language QASM and host languages such as Python and JavaScript in various quantum computing frameworks [18] such as Qiskit (https://qiskit.org/documentation/getting_started.html accessed on 15 June 2023), ProjectQ (https://projectq.ch/ accessed on 15 June 2023), and Pennylane (https://pennylane.ai/ accessed on 15 June 2023).

#### 2.2.2. Quantum Neural Network

**Figure 3:**
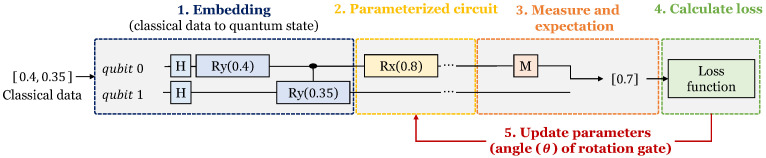
Training process of quantum neural network.

A quantum neural network [19] is an artificial intelligence that utilizes quantum mechanics phenomena (entanglement and superposition). Figure 3 shows the training process of a quantum neural network. A quantum neural network consists of qubits and quantum gates on a quantum computer. Therefore, it learns quantum state data by encoding classical data into quantum data. There are several types of data encoding: basis embedding, angle embedding, amplitude embedding, etc. Through the data embedding process, classical data are converted into a quantum state that quantum circuits can learn. After the embedding process is completed, a parameterized quantum circuit consisting of a rotating gate with parameters is executed. The state of each qubit changes as the circuit is performed, and the state of the qubit is measured at the end of the circuit. Based on this measured value, the loss is calculated and the rotation angle of the quantum gate is updated. Then, the quantum circuit to which the changed parameters are applied is re-executed. By repeating this process the quantum neural network is trained.

Quantum neural networks require fewer parameters and fewer training data than classical neural networks. They also have the advantage of being able to perform better than classical neural networks. However, with current quantum computers, it is difficult to use many qubits [20]. Therefore, the quantum–classical hybrid neural network [21,22] combined with the classical neural network is now widely used. In a quantum–classical hybrid neural network, a parameterized quantum circuit is used as one of the layers of a classical neural network. Therefore, elements such as loss functions, optimization functions, metrics, and epochs are used the same as in classical neural networks. Currently, hybrid neural networks have the advantage of being more stable and able to obtain higher accuracy than using only quantum circuits. However, in NISQs, it is difficult to exceed the performance of classical neural networks for complex problems. Currently, there are many studies finding quantum advantages. However, there are opinions that when identifying the tasks that quantum neural networks can solve and analyze, both the positive and negative results should direct the research for the quantum neural networks [23] (https://discuss.pennylane.ai/t/why-quantum-circuit-in-deep-learning/426 accessed on 15 June 2023, https://discuss.pennylane.ai/t/qml-algorithm-doesnt-learn/468/21 accessed on 15 June 2023). Despite these limitations, quantum neural network models with various structures are currently being studied [24,25,26,27,28,29,30], and most of them are used in a hybrid way.

### 2.3. Differential Characteristics

Differential cryptanalysis [31] is a representative cryptanalysis method of block ciphers. The input difference (δ) is the XOR between the plaintext pairs ($P_{0},P_{1}$), and the output difference (Δ) is the XOR between the ciphertext pairs. That is, as in Equation (1), if the delta is XORed to the random plaintext, it is calculated as $P_{1}$. Furthermore, the results of encrypting (*E*) $P_{0}$ and $P_{1}$ are $C_{0}$ and $C_{1}$, respectively. Finally, by XORing $C_{0}$ and $C_{1}$, the output difference (Δ) can be calculated. A pair of input and output differences ((δ,Δ)) is called a differential. If encrypted data can be distinguished from random using the differential characteristic, data complexity is significantly lower than that of an exhaustive search. In the case of an ideal encryption algorithm, when plaintext with any input difference is encrypted, the output difference should be uniform. Conversely, a weak cipher has a certain output difference. If the probability of having an output difference for an input difference is greater than the random probability, the ciphertext can be distinguished from the random. These characteristics are propagated even when encryption is performed and can be predicted probabilistically.

(1) $$\begin{array}{c} P_{1}=P_{0}⊕\delta ,\\ C_{0}=E\left(P_{0}\right),C_{1}=E\left(P_{1}\right),\\ \Delta =C_{0}⊕C_{1} \end{array}$$

### 2.4. Neural-Network-Based Distinguisher for Differential Cryptanalysis

#### 2.4.1. Basic Design of Neural Distinguisher

**Figure 4:**
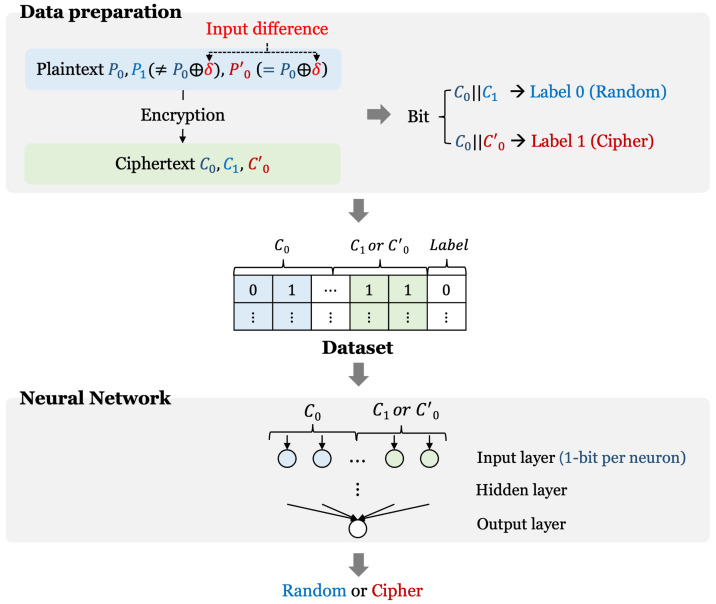
Basic design of neural distinguisher.

The neural distinguisher performs an attack that distinguishes cryptographic data from random data by utilizing deep learning networks and differential characteristics. Figure 4 shows the overall process of a basic neural distinguisher. The most basic model has a structure that distinguishes between random data and cryptographic data satisfying one differential characteristic. Therefore, it is necessary to generate a random ciphertext pair and a cryptographic data pair satisfying one differential characteristic. The paintext pair ($P_{0}$, $P_{0}^{\prime }$) with an input difference by XORing one input difference (δ) with random plaintext ($P_{0}$) can be created. Then, when this plaintext pair is encrypted, a ciphertext pair ($C_{0}$, $C_{0}^{\prime }$) that probabilistically satisfies the output difference is generated. A random data pair ($C_{0}$, $C_{1}$) can be obtained by encrypting two random plaintexts ($P_{0}$, $P_{1}$). A data set is generated by labeling these random and cryptographic data pairs as 0 and 1, respectively. Next, the data set is fed into the neural network. At this time, 1 bit of the data set is allocated to each neuron. The neural network learns the characteristics of the input data. As a result, the neural distinguisher can distinguish cryptographic data pairs from random pairs. This is the basic neural distinguisher architecture, and there are several architectures: using multiple types of single input differential characteristics, using multiple input differential characteristics, using multiple input differential combinations, etc.

#### 2.4.2. Related Works

At CRYPTO 2019, Gohr [4] proposed the first deep-learning-based neural distinguisher for round-reduced SPECK. They used a deep-learning-based classifier to distinguish random data from input differences, and reduced data complexity for key recovery attacks compared to a classical distinguisher. The accuracy of the neural network distinguisher for 8-round SPECK is about 0.514, and since we obtained an accuracy of 0.5 or more, we can classify cipher data from random data. After Gohr’s work, in [8], differential cryptanalysis for SIMON was performed using the deep-learning-based neural distinguisher. In [4], one ciphertext pair (two ciphertexts) was used as input data, whereas *k* (k>1) ciphertext pairs were used as input data in [5]. That is, *k* ciphertext pairs are input to the neural distinguisher and classified. Their target ciphers are SPECK, CHASKEY, PRESENT, and DES. The experiment was conducted according to the *k*, and an accuracy of 0.5 or more was obtained in all cases. In [6], they conducted experiments on multiple input differentials in 5-round SPECK-32. It was analyzed that there are differential characteristics with a higher probability than 0x00400000, which is the input difference of the 5-round SPECK used in Gohr’s work. They experimented with the top 25 input differences. In most cases, an accuracy of 75% or better was achieved, and for 0x28000010, an accuracy of 90% or more was achieved. In [7], the target ciphers are GIMLI, ASCON, KNOT, and CHASKEY, and two models were proposed considering multiple input differences and single difference. The neural distinguisher for multiple input differences performs multi-classification by setting each input difference as a class, and the neural distinguisher for single input difference performs binary classification on random data and ciphertext data. Baksi et al. experimented using MLP, CNNs, and LSTM (Long Short-Term Memory) for GIMLI-CIPHER. In this task, MLP performed the best, and CNN could not achieve an accuracy higher than 0.5. Therefore, in their work, the MLP model was used for other ciphers. However, the existing deep-learning-based neural distinguisher has limitations in round expansion due to lack of memory and data complexity. To overcome this, a new approach combining a classical distinguisher and neural distinguisher has been proposed in [9]. Input the input difference to the classical distinguisher to find the output difference for *r* rounds. Then, the distinguisher for the extended round was proposed, using the output difference of the *r* round as the input difference of the neural distinguisher. As such, research on various ciphers, input differences, neural network structures, etc., is being actively conducted using neural distinguishers, and current studies are the result of round-reduced ciphers, not full rounds.

## 3. Design of Quantum Neural Distinguisher for SPECK-32

In this paper, we present a quantum neural distinguisher using a quantum–classical hybrid neural network for SPECK-32. The purpose of a neural distinguisher is to distinguish the ciphertext pair from the random pair using differential characteristics. Propagation of the differential characteristics to the nonlinear layer is probabilistically predictable even if encryption is repeated, and the cipher data used have differential characteristics. Therefore, by using neural networks, data with differential characteristics can be distinguished from random data. Quantum–classical hybrid neural networks also perform training and prediction for data, so they can distinguish the cipher from random.

**Figure 5:**
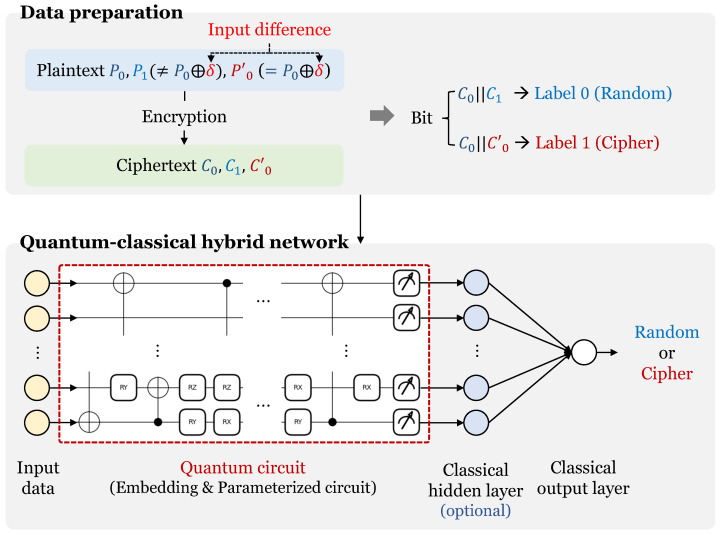
Diagram of the proposed method.

Figure 5 shows the system diagram of the proposed quantum neural distinguisher. Our method has two steps (data preparation and training of a quantum–classical hybrid neural network). Data preparation is the process of generating data with differential characteristics and random data for training. In addition, the quantum–classical hybrid neural network is composed of quantum circuits and classical layers as described above. Since the quantum–classical neural network can learn the characteristics of data like the classical neural network, it can distinguish the cipher data from random. So, it can be used as a distinguisher. Details of both parts are described below.

### 3.1. Data Set Preparation

First, two random plaintexts ($P_{0}$, $P_{1}$) are selected. These two plaintexts do not satisfy the input difference (δ = 0x00000040). Then, the input difference is XORed to one random plaintext ($P_{0}$); this XORed value is called $P_{0}^{\prime }$. After these three plaintexts are generated, each plaintext is encrypted with SPECK-32. These are called $C_{0},C_{1}$, and $C_{0}^{\prime }$, respectively. $C_{0}$ and $C_{1}$ are random ciphertext pairs because they are encrypted values of unrelated random plaintext pairs. Furthermore, $C_{0}$ and $C_{0}^{\prime }$ are ciphertext pairs obtained by encrypting plaintext pairs that satisfy the input difference. Thus, a random ciphertext pair is labeled class 0, and a differential ciphertext pair is labeled class 1. When this process is completed for all data, the data set required to train the quantum neural network based distinguisher is created. The data set generated through this process is input to the quantum–classical hybrid neural network.

### 3.2. Structure of Quantum Neural Distinguisher

#### 3.2.1. Overall Network Structure

**Figure 6:**
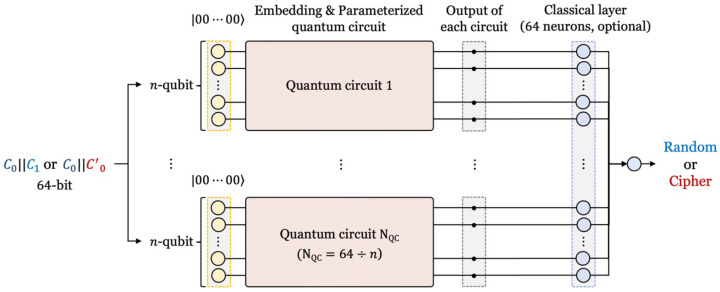
Overall structure of quantum neural distinguisher.

Figure 6 shows the detailed architecture of a quantum–classical hybrid neural network. The input of the hybrid neural network are cipher pairs or random data pairs ($C_{0}\left|\right|C_{0}^{\prime }$ or $C_{0}\left|\right|C_{1}$) generated in the data preparation process. Since the block size of SPECK-32, the target cipher, is 32-bit, the data input to the network are 64-bit and have 64 data features. In classical neural networks, 1 bit of input data is allocated to 1 neuron. In this work, 1 bit of the input data is allocated to 1 qubit. However, this does not require 64 qubits. Since many qubits cannot be used in current quantum computing, input data are divided. The 64-bit data are divided by *n*, then input to 64÷n quantum circuits with *n* qubits. If *n* is 8, 64-bit data are divided into 8 bits. Then each 8-bit data segment is input into 8 quantum circuits. Therefore, 64 qubits are not required, and the quantum circuit can be run with only *n* qubits. After the data of *n*-bit is input to the quantum circuit with *n*-qubit, data embedding and parameterized quantum circuit are executed. At this time, the initial values of the qubits of the quantum circuit are set to zero. At the end of the quantum circuit, the value of each qubit is measured as *n* classical values. The measured value can optionally be input to the classical hidden layer or can be directly input to the output layer. The output layer is a classical layer, and it classifies randoms and ciphers.

Since our quantum-classical neural distinguisher is a quantum–classical hybrid network, the loss function and optimization function used are classical methods. Since it is a binary classification, the binary cross-entropy loss function is used, and the activation function of the output layer is sigmoid. Furthermore, the Adam optimization function is used. In addition, the adjoint differentiation (https://pennylane.ai/qml/demos/tutorial_adjoint_diff.html accessed on 15 June 2023) was used to calculate the parameters of the quantum circuit, such as calculating the gradient in the classical neural network. This is a time- and memory-efficient method compared to other methods (i.e., parameter shift, backprop). We use the adjoint method with an accelerated simulator to simulate more quantum resources and increase the training speed.

#### 3.2.2. Data Embedding

**Figure 7:**
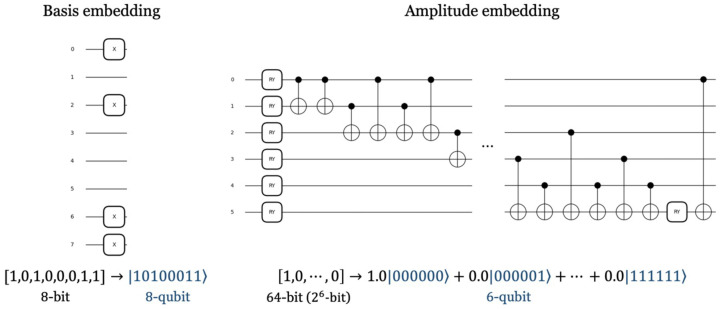
Quantum circuits for data embedding.

As explained above, 64-bit data are input to quantum circuits after being divided. Quantum circuits extract and learn data features like hidden layers in classical neural networks. Prior to training, data are required in a form that the quantum circuit can process. Thus, classical data must be encoded into quantum states. There are various embedding methods. Among them, Figure 7 shows two embedding schemes for representing classical data as quantum data. Basis embedding represents each classical bit through each qubit. First, all qubits are set to a state of 0. If the classical bit to be encoded is 1, an X gate is applied to the corresponding qubit. Then, the state of the qubit to which the X gate is applied is changed to 1. This method is suitable for representing binary data. *n*-qubits are required to represent *n*-bit data, but the circuit for embedding has low depth and is simple. In our work, we encode the input data, not the weights of the neural network. For this, a basis embedding suitable for embedding cryptographic data was used. Amplitude embedding is a method of expressing classical data as an amplitude vector of qubits and is suitable for continuous and normalized data. Since the length of the amplitude vector for *n*-qubits is $2^{n}$, data of $2^{n}$-bit can be embedded using only *n*-qubits. Since it requires a relatively small number of qubits, it is easy to embed many data into a single quantum circuit, but it is more complex and has a higher depth than basis embedding.

#### 3.2.3. Parameterized Quantum Circuit

**Figure 8:**
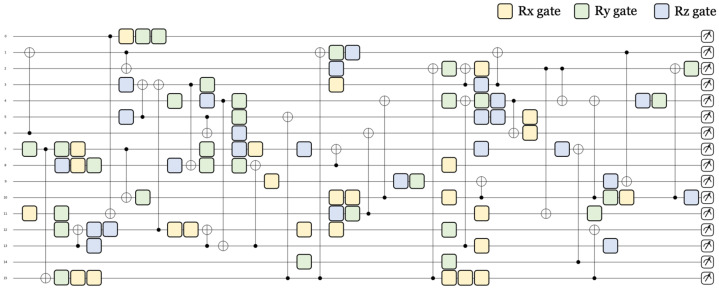
Randomly parameterized quantum circuit with 16 qubits.

After embedding data into quantum states, quantum gates with parameters are applied to qubits. The state of the qubits changes according to the parameter of the quantum gates [32,33,34,35,36,37]. Depending on the state of the qubit, the predicted value of the neural network changes. That is, quantum circuits can change the state of qubits with parameters. Then, training is performed by changing the state of the parameters of the circuit so that the correct result can be inferred in the quantum circuit. Figure 8 shows the parameterized quantum circuit diagram used in the quantum neural distinguisher. Rx, Ry, and Rz gates are used, and by rotating based on the x, y, and z axes, all values on the Bloch sphere can be obtained. Through this, it becomes a superposition state and, unlike classical bits, can have a wider range of values. In addition, through CNOT, a 2-qubit gate, several qubits become entangled and affect the states of another qubit. The construction of parameterized circuits for learning is similar to the way neurons in classical neural networks are connected to each other with weights (parameters).

Parameterized quantum circuits are not constructed according to specific rules. Quantum circuits with high performance can be constructed without any rules, and Pennylane provides several templates to easily create parameterized quantum circuits. In our work, the ’Random layer’ was used. Random parameterized quantum circuits provided by Pennylane can be made by stacking quantum layers. As quantum layers are stacked, quantum gates are added to the circuit and the depth of the circuit increases. In addition, the rotation gate and entanglement of the circuit are randomly set according to the number of quantum layers and the number of qubits. At the end of the quantum circuit, measurements are performed on each qubit. Through this, the final state of each qubit is determined as a classical value, and measured values, numbering as many as the number of qubits, are calculated in each circuit. Through measurements on all circuits output 64 classical values, which are passed to the output layer. That is, in the output layer, prediction is performed based on the output value of the quantum circuit.

## 4. Experiment and Evaluation

For the experiment, we used an Intel i7-13700KF CPU with 32GB RAM and an NVIDIA GeForce RTX 4080 with 16GB RAM on Ubuntu 20.04.5 LTS. In terms of the programming environment, Python 3.9, Tensorflow 2.9.0, and Pennylane [22] (a library for hybrid neural networks) were used. As a simulator for performing quantum circuits, a C++-based acceleration simulator (lightning.qubit) provided by Pennylane was used.

The performance (accuracy) of quantum neural networks is determined by the connectivity and expressiveness of quantum circuits [38]. Experiments were conducted and evaluated in terms of factors related to connectivity and expressiveness: number of qubits, embedding method, and number of quantum layers.

### 4.1. Numerical Experiments and Results

In this section, we conduct numerical experiments on factors that affect performance. However, on the NISQ, it took 3500 to 7500 s for 1 epoch depending on the case, so many experiments could not be conducted. Therefore, we conducted more than 3 experiments for each element and discussed based on the results. We plan to conduct more and more diverse experiments in this regard in future work.

#### 4.1.1. Accuracy According to Data Embedding

Table 1 shows the accuracy according to the data embedding method. We used the same parameterized quantum circuit for both embedding schemes and set the number of quantum layers to 5 and the epochs to 10. Furthermore, we used 16 qubits, which is the maximum number of qubits available in the current quantum computing environment.

Amplitude embedding requires a small number of qubits and can embed data into a single quantum circuit. However, considering the characteristics of the data to be trained, this is not recommended. As a result of the experiment, when using amplitude embedding, the test accuracy was 0.5, so it was impossible to distinguish the input data. Since the random probability is 0.5, the training and test accuracy must be at least 0.51 to distinguish the cipher data from random data. In general, if accuracy exceeds 0.51, it is considered valid. Because, if it does not exceed 0.51, it has 50% accuracy (e.g., 50.1%, 50.5%, etc.). Therefore, we set the distinguisher to be valid when the accuracy is greater than 0.51. In addition, the distinguisher does not perform a differential attack directly, but it reduces data complexity in a differential attack (e.g., last round attack [39]). Therefore, our implementation can be used as a distinguisher even if it is not highly accurate. In other words, although the reliability is not high, it can be said to be a valid distinguisher. In contrast, when basis embedding was used, it exceeded 0.51 in all test cases (We used a test data set consisting of $2^{9.9660}$ rather than a single data point; furthermore, the calculated accuracies are averages over three experiments.) From this result, it can be seen that basis embedding is appropriate for the quantum neural distinguisher.

#### 4.1.2. Accuracy According to the Number of Quantum Layers

Table 2 shows the result according to the number of quantum layers. We used the random parameterized circuit provided by Pennylane. As a result of the experiment, when using a quantum circuit with 10 layers, it was possible to learn from the data, and the accuracy was higher than when using a parameterized quantum circuit with 5 layers. However, the inference performance deteriorated due to overfitting, and the test accuracy did not exceed 0.51. On the other hand, when using 5 quantum layers, a test accuracy of 0.51 or higher was obtained for all test cases, and overfitting did not occur. As one quantum layer is added, the quantum parameter increases by the number of qubits, so a parameterized quantum circuit with 10 quantum layers has a higher circuit depth and twice the number of quantum parameters than a circuit with 5 layers. That is, as the number of quantum layers increases, the number of parameters per single qubit increases (see Table 3). The factor that increases the number of parameters includes the number of qubits, but the greater the number of qubits for the same number of quantum parameters, the smaller the parameter for a single qubit. As in classical neural networks, overparameters cause overfitting, and in this work, when the number of parameters per single qubit exceeds 5, it becomes an overparameter state. Therefore, when using Pennylane’s random circuit with 16 qubits, if more than 5 layers are used, there is a risk of performance deterioration due to overparameters. If the number of parameters must be increased to increase the capacity of the network, increasing the number of qubits will be better because it does not cause overparameterization. However, the increased circuit depth and connectivity by adding qubits may not be suitable to run on an NISQ, so proper tuning is required.

#### 4.1.3. Accuracy According to the Number of Qubits

Table 4 shows the accuracy according to the number of qubits. Compared to a circuit with 8 qubits, a quantum circuit with 16 qubits has more quantum gates, a larger number of quantum parameters, and a larger circuit depth and circuit size. That is, the complexity and the number of parameters of the circuit increase. However, this is sufficiently available in an NISQ and is not an overparameter state considering the number of parameters per single qubit. In addition, connectivity increased by adding qubits, and higher test accuracy was achieved due to higher connectivity. In particular, when using an 8-qubit circuit, there were some cases where the test accuracy did not exceed 0.5. However, when using quantum circuits with 16 qubits, an accuracy of better than 0.51 was achieved in all cases. As such, it was confirmed that increasing the number of qubits increases the connectivity of the circuit and improves performance as more data points are reflected in a single circuit. Since there are many factors such as circuit complexity and data complexity, it is not certain that the greater the number of qubits, the better. However, considering the above experimental results, it is thought that it would be good to increase the number of qubits for this work if more qubits become operable in the future quantum computer.

### 4.2. The Best Model of Our Quantum Neural Distinguisher

**Figure 9:**
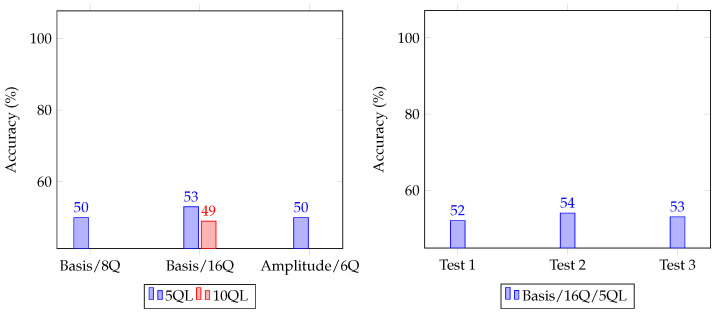
Accuracy of our quantum neural distinguishers depending on options (**left side**), XXX (**right side**).

Figure 9 shows the accuracy of our quantum neural distinguishers depending on options (left side), and the accuracy of each test data set for our best model (right side). As mentioned above, we conducted three experiments using training and test data sets due to the very long training time. Q and QL are the number of qubits and quantum layers, respectively, (e.g., 5Q means 5 qubits. It can be confirmed that our final model (Basis/16Q/5QL), constructed through our experiments, exceeds the accuracy of 0.51 for all test data sets for the 5-round SPECK-32.

### 4.3. Comparison of Quantum and Classical Neural Distinguisher

In this section, we compared our quantum neural distinguisher with classical methods. In this experiment, three approaches were considered due to the limited environment of quantum neural networks (quantum resources, memory, and time limitations). The first (only Q) is the method used in the above experiments, using only a quantum circuit as a hidden layer. The second (constrained C) is a neural network that uses only 1 classical hidden layer, and uses the same limited data size, epoch, and batch size as the quantum methods. The last approaches (C1 and C2) are the results presented in [4] and [5], respectively. The last method works for more than 5 rounds, but for comparison, we compared the accuracy for 5 rounds.

Table 5 shows the comparison of quantum and classical neural distinguisher for 5-round SPECK-32. C1 [4] and C2 [5] without data and parameter restrictions achieved higher accuracy than others, and the constrained C showed better performance than only Q. Furthermore, as we saw earlier, the only Q method using 16 qubits was able to distinguish 5 rounds. As such, it is currently difficult to obtain quantum advantages for complex data in NISQs.

However, our approach can be used as a quantum neural distinguisher in NISQs despite the constrained environment, and we were able to confirm its potential. To improve the accuracy of the only Q method, it would be necessary to be able to embed more data into a single circuit and increase the number of training data. In addition, it is believed that the number of parameters across the network is insufficient, and the number of parameters should be increased without excessively increasing the number of parameters per single qubit. Several methods could be used to increase the number of parameters, but basically more qubits would have to be available. If more resources and more efficient learning techniques are developed in the future, we expect to achieve better accuracy as more data and resources become available.

### 4.4. Quantum Neural Distinguisher in NISQ

Quantum neural distinguishers in NISQs have many limitations including the number of data/parameters and training time.

Currently, real QPUs are not easy to use, and large numbers of qubits are not available. Therefore, we have to use the simulator in general. When using a simulator, 1 epoch takes between 3500 and 7500 s depending on the device used (here, the number of data used is only 15,000 training data). According to our experiments, if the number of data increases *n* times, the training time also increases *n* times in the Pennylane simulator. Therefore, we cannot realistically use a large number of data. In fact, compared to $2^{23.5234}$ training data that can be used in classical neural networks, the number of data available in quantum neural networks is significantly smaller. In (quantum and classical) deep learning, the number of training data is a very important factor affecting accuracy. Therefore, it is difficult for the quantum neural distinguisher to outperform the classical neural distinguisher in an NISQ. In addition, the number of parameters is also a constraint in NISQs. The larger the number of parameters, the longer the training time. Therefore, a limited number of parameters must be used to train in a feasible time.

Due to these limitations, it is difficult to achieve high performance of a quantum neural distinguisher in NISQs. It is thought that the performance will improve when simulators and technologies for accelerating training are developed, or a way to use more quantum resources in a reasonable time is presented.

## 5. Conclusions and Future Work

Although many studies are being conducted to find various problems that quantum artificial intelligence can solve, an NISQ’s quantum neural network technology is difficult to use to obtain quantum advantages for complex data. In this work, we proposed a distinguisher based on a quantum neural network for SPECK-32. As a result of the experiment, the classical method achieved an accuracy of 0.93, our quantum neural distinguisher achieved an accuracy of 0.53 due to limitations in time, data, and parameters, and the constrained classical method (under the same limited conditions as the quantum method) achieved an accuracy of 0.76. Our target cipher, SPECK-32, has 32-bit plaintext, so they are complex data with a total of 64 data points, and as shown in the experimental results, it is still difficult to obtain quantum supremacy. However, we confirmed that our work is feasible in an NISQ and can operate as a distinguisher for 5-round SPECK-32. We evaluated our implementation in terms of the number of qubits, the embedding scheme, and the number of quantum layers, and compared it with the classical method. As a result, the basis embedding was more suitable for the quantum neural distinguisher. In addition, since adding a quantum layer for higher expressiveness can greatly increase the depth of a circuit or cause overparameters, it is thought to be better to increase the number of qubits to increase network capacity. However, increasing the number of qubits also increases the complexity of the circuit, so extending a quantum circuit does not guarantee performance improvement. Therefore, a tuning process is needed to properly set all elements in consideration of connectivity and expressiveness. In the future, recently developed technologies such as data re-uploading [30] or circuit cutting should be applied so that more quantum resources, data, and time can be used. Furthermore, we will consider different kinds of embedding schemes, such as trainable embeddings [40], for better results. In addition, since quantum neural network technology is developing day by day, it is thought that there will be another suitable methodology for distinguishers. Furthermore, the analysis of the various factors presented in this paper is expected to help design new approaches to achieve better performance in the future.

## Figures and Tables

**Table 1:** Result according to the data embedding method (The number of quantum layers: 5, Epoch: 10).

Embedding Method	Basis	Amplitude
Qubits	16	6
Training accuracy	0.52	0.51
Test accuracy	0.53	0.50

**Table 2:** Results according to the number of quantum layers (using basis embedding, Epoch: 10).

Quantum layer	5	10
Qubit	16	
Depth	22	42
Quantum parameters	80	160
Training accuracy	0.52	0.53
Test accuracy	0.53	0.49

**Table 3:** The number of quantum parameters depending on the number of qubits and quantum layers.

Qubit	Quantum Layers	Quantum Parameters	Parameters Per Single Qubit
8	5	40	5
8	10	80	10
16	5	80	5
16	10	160	10

**Table 4:** Result according to the number of qubits (using basis embedding, number of quantum layers: 5, Epoch: 10). Since there is a connection between one qubit and the qubits it affects, we called the average number of entangled (affected) qubits per single qubit the circuit’s connectivity in this experiment.

Qubits	8	16
Gates (Rx,Ry,Rz,CNOT)	12, 16, 12, 16	26, 29, 25, 31
Quantum parameters	40	80
Depth	14	22
Quantum layers	5	5
Quantum circuit size	112	352
Connectivity	5.00	7.93
Training accuracy	0.52	0.52
Test accuracy	0.50	0.53

**Table 5:** Comparison of quantum and classical neural distinguisher for 5-round SPECK-32.

Method	Only Q	Constrained C	C1 [4]	C2 [5]
Qubits	16	-		
Quantum layers	5	-		
Embedding method	Basis	-		
Parameters	385	4353	100897	Can be adjusted adaptively
Data size (Training, Test)	$2^{13.8727}$, $2^{9.9660}$		$2^{23.2534}$, $2^{19.9316}$	
Epoch	10		200	10
Batch size	32		5000	500
Training accuracy	0.52	0.79	0.93	0.99 (Max)
Test accuracy	0.53	0.76	0.93	0.99 (Max)

## Data Availability

Source codes of proposed method are available in https://github.com/khj1594012/quantum-neural-distinguisher-for-speck32 accessed on 15 June 2023.
